# Crystal structures of morpholinium hydrogen bromanilate at 130, 145 and 180 K

**DOI:** 10.1107/S2056989015017272

**Published:** 2015-09-26

**Authors:** Kazuma Gotoh, Yuki Tahara, Hiroyuki Ishida

**Affiliations:** a Department of Chemistry, Faculty of Science, Okayama University, Okayama 700-8530, Japan

**Keywords:** crystal structure, bromanilic acid, morpholine, hydrogen-bonding, proton disorder

## Abstract

Crystal structures of the title compound (systematic name: morpholin-4-ium 2,5-di­bromo-4-hy­droxy-3,6-dioxo­cyclo­hexa-1,4-dien-1-olate), C_4_H_10_NO^+^·C_6_HBr_2_O_4_^−^, were determined at three temperatures, *viz.* 130, 145 and 180 K. The asymmetric unit comprises one morpholinium cation and two halves of crystallographically independent bromanilate monoanions, which are located on inversion centres. The conformations of the two independent bromanilate anions are different from each other with respect to the O—H orientation. In the crystal, the two different anions are linked alternately into a chain along [211] through a short O—H⋯O hydrogen bond, in which the H atom is disordered over two positions. The refined site-occupancy ratios, which are almost constant in the temperature range studied, are 0.49 (3):0.51 (3), 0.52 (3):0.48 (3) and 0.50 (3):0.50 (3), respectively, at 130, 145 and 180 K, and no significant difference in the mol­ecular geometry and the mol­ecular packing is observed at the three temperatures. The morpholinium cation links adjacent chains of anions *via* N—H⋯O hydrogen bonds, forming a sheet structure parallel to (-111).

## Abstract 2

Crystal structures of morpholinium hydrogen bromanilate have been determined at 130, 145 and 180 K. The asymmetric unit comprises one morpholinium cation and two halves of crystallographically independent bromanilate monoanions. The conformations of the two independent bromanilate anions are different from each other with respect to the O—H orientation. The two different anions are linked alternately into a chain though a short O—H⋯O hydrogen bond, in which the H atom is disordered over two positions.

## Chemical context   

Anilic acid (2,5-dihy­droxy-1,4-benzo­quinone) derivatives, such as chloranilic acid (2,5-di­chloro-3,6-dihy­droxy-1,4-benzoqinone) and bromanilic acid (2,5-di­bromo-3,6-dihy­droxy-1,4-benzoqinone), appear particularly attractive as a versatile template for generating hydrogen-bonded self-assemblies with various organic bases (Zaman *et al.*, 2001 ▸; Molčanov & Kojić-Prodić, 2010 ▸; Gotoh & Ishida, 2011 ▸; Thomas *et al.*, 2013 ▸) and also as a model compound for investigating proton dynamics in hydrogen-bond systems (Ikeda *et al.*, 2005 ▸; Seliger *et al.*, 2009 ▸). Furthermore, salts and co-crystals of anilic acids with organic bases have attracted much inter­est with respect to organic ferroelectrics (Horiuchi *et al.*, 2008 ▸, 2009 ▸, 2013 ▸).

In our previous study, we reported the crystal structure of morpholinium hydrogen chloranilate, C_4_H_10_NO^+^·C_6_HCl_2_O_4_^−^, in which a short O—H⋯O hydrogen bond is formed between the chloranilate ions and the H atom in the hydrogen bond is disordered over two sites (Ishida & Kashino, 1999 ▸). The measurements of ^35^Cl NQR (nuclear quadrupole resonance) for the compound in the temperature range 4–300 K showed an anomalous temperature dependence of the NQR frequencies, which cannot be explained by the conventional Bayer-type lattice motion: one of the two frequencies exhibits an anomalous increase with increasing temperature from 4.2 K while the other frequency shows a rather fast decrease with temperature. The anomalous behavior was ascribed to a drastic temperature variation of the disordered O—H⋯O hydrogen bond, as revealed by multi-temperature X-ray diffraction (Tobu *et al.*, 2012 ▸). In the present study, we have undertaken the structural determination of morpholinium hydrogen bromanilate, C_4_H_10_NO^+^·C_6_HBr_2_O_4_^−^, to extend the study of hydrogen-bonding in the amine-halo­hydroxy­benzo­quinone system.
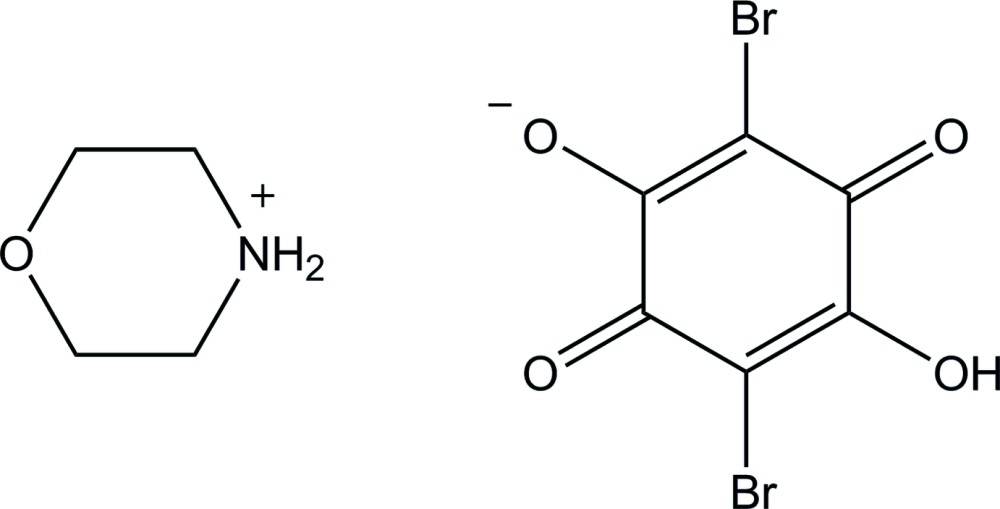


## Structural commentary   

**Figure 1:**
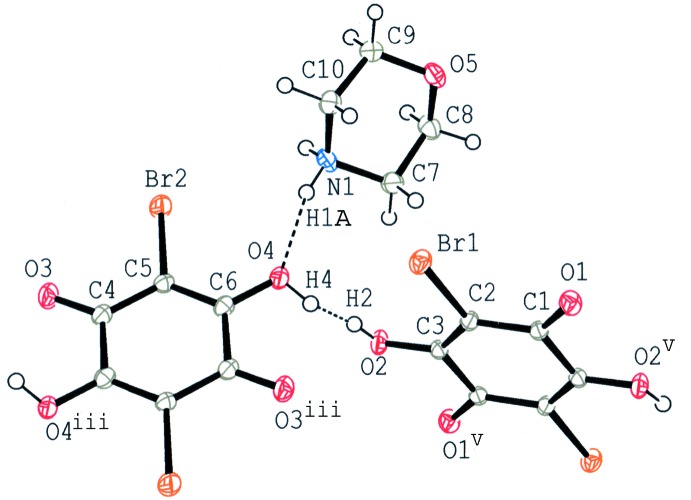
A view of the mol­ecular structure of the title compound at 180 K, showing the atom-numbering scheme. Displacement ellipsoids of non-H atoms are drawn at the 50% probability level and H atoms are drawn as circles of arbitrary size. The site-occupancy factors of the disordered H atom (H2 and H4) are approximately equal. The N—H⋯O and O—H⋯O hydrogen bonds are indicated by dashed lines. [Symmetry codes: (iii) −*x*, −*y* + 1, −*z*; (v) −*x* + 2, −*y* + 2, −*z* + 1.]

**Figure 2:**
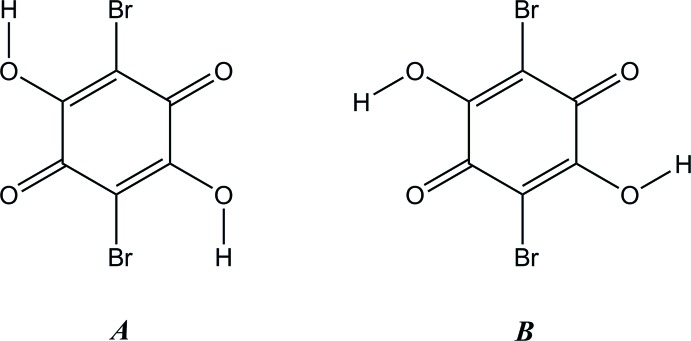
Two conformations (*A* and *B* forms) of bromanilic acid with respect to the O—H orientation.

The title compound is isomorphous with morpholinium hydrogen chloranilate in the space group *P* 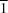 (Ishida & Kashino, 1999 ▸; Tobu *et al.*, 2012 ▸) and has a quite similar mol­ecular packing to the chloranilate. The asymmetric unit of the title compound comprises one morpholinium cation and two halves of crystallographically independent bromanilate monoanions, which are each located on an inversion centre (Fig. 1 ▸). The conformations of two bromanilate anions are different from each other with respect to the O—H orientation as shown schematically in Fig. 2 ▸.

In morpholinium hydrogen chloranilate, the bond distances of C3—O2 and C6—O4, which are involved in the disordered O—H⋯O hydrogen bond, showed slight but systematic decrease and increase, respectively, with temperature [C3—O2: from 1.2994 (10) Å at 114 K to 1.2951 (10) Å at 180 K; C6—O4: from 1.290 (10) Å at 114 K to 1.2946 (10) at 180 K], which corresponds to population changes of the two disordered proton sites in the hydrogen bond (Tobu *et al.*, 2012 ▸). In the present compound, however, the C3—O2 and C6—O4 bond lengths are almost constant [C3—O2: 1.2953 (17), 1.2937 (17) and 1.2931 (17) Å at 130, 145 and 180 K; C6—O4: 1.3002 (18), 1.2997 (18) and 1.2997 (18) Å at 130, 145 and 180 K] and no significant difference in the mol­ecular geometry is observed at the three temperatures.

## Supra­molecular features   

**Figure 3:**
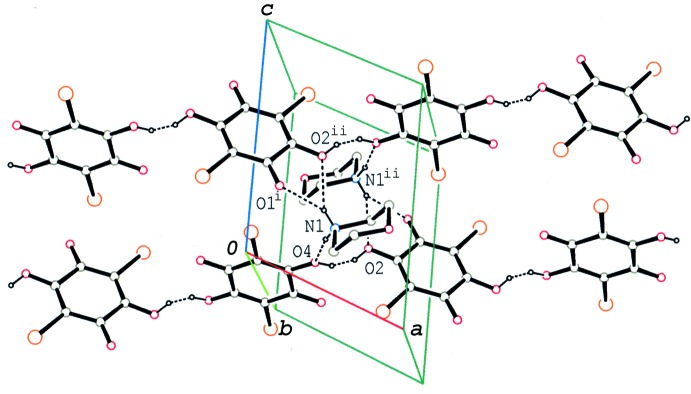
A partial packing diagram of the title compound at 180 K, showing the hydrogen-bonded aggregate of morpholinium and hydrogen bromanilate ions. The N—H⋯O and O—H⋯O hydrogen bonds are indicated by dashed lines. [Symmetry codes: (i) *x* − 1, *y* − 1, *z*; (ii) −*x* + 1, −*y* + 1, −*z* + 1.]

**Figure 4:**
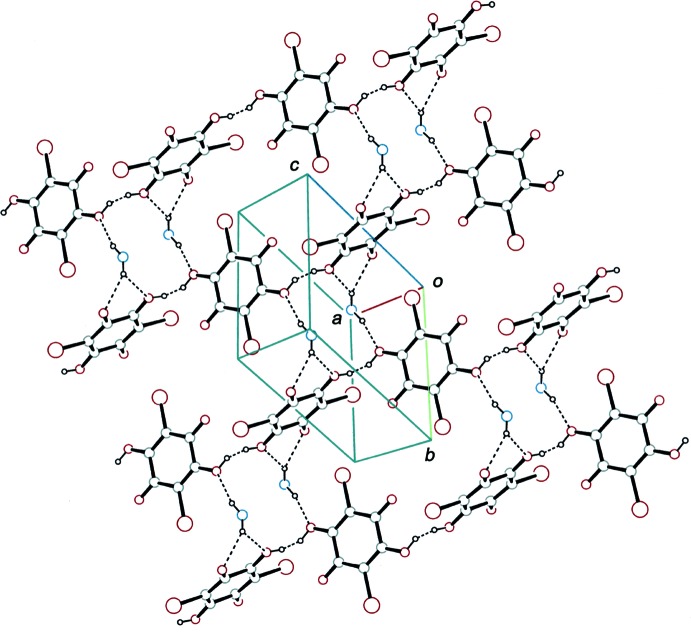
A packing diagram of the title compound at 180 K, showing the sheet structure formed through N—H⋯O and O—H⋯O hydrogen bonds (dashed lines). For the morpholinium cations, only NH_2_ groups are shown for clarity.

In the crystal, the two independent bromanilate anions with different conformations are linked alternately by short O—H⋯O hydrogen bonds (Tables 1 ▸, 2 ▸ and 3 ▸), forming a chain along [211] (Fig. 3 ▸). The adjacent independent anions are almost perpendicular to each other, with dihedral angles of 86.57 (7)° (130 K), 86.65 (7)° (145 K) and 86.81 (7)° (180 K) between the benzo­quinone rings. The morpholinium cation connects the anion chains through N—H⋯O hydrogen bonds and a weak C—H⋯O hydrogen bond into a sheet parallel to (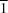
11) (Fig. 4 ▸). Between the chains, short Br⋯O and Br⋯C contacts [Br2⋯O1^i^: 3.1698 (13) Å (130 K), 3.1725 (13) Å (145 K) and 3.1763 (13) Å (180 K); Br2⋯C1^i^: 3.2673 (15) Å (130 K), 3.2716 (15) Å (145 K) and 3.2808 (15) Å (180 K); symmetry code: (i) *x*- 1, *y* − 1, *z*] are observed. A weak C—H⋯Br inter­action is also observed between the sheets.

## Database survey   

Although a search of the Cambridge Structural Database (Version 5.36, last update February 2015; Groom & Allen, 2014 ▸) for organic salts and co-crystals with bromanilic acid gave 31 hits, no crystal structure including the *A* form (Fig. 2 ▸) was found.

## Synthesis and crystallization   

Single crystals of the title compound suitable for X-ray diffraction were prepared by slow evaporation from an aceto­nitrile solution (200 ml) of bromanilic acid (200 mg) with morpholine (60 mg) at room temperature.

## Refinement   

Crystal data, data collection and structure refinement details are summarized in Table 4 ▸. C-bound H atoms of the morpholinium cation were positioned geometrically with C—H = 0.99 Å and were refined as riding with *U*_iso_(H) = 1.2*U*_eq_(C). The N-bound H atom was located in a difference Fourier map and refined freely [refined N—H = 0.85 (3)–0.89 (3) Å]. Two disordered positions of the H atom in the O—H⋯O hydrogen bond were located in a difference Fourier map. Since site occupancy factors and isotropic displacement parameters are correlated and bonding effects also make the site-occupancy factors less reliable, the positional parameters and the occupancies of the H atom were refined, with *U*_iso_(H) = 1.5*U*_eq_(O), and with distance restraints of O—H = 0.84 (2) Å.

## Supplementary Material

Crystal structure: contains datablock(s) General, 1, 2, 3. DOI: 10.1107/S2056989015017272/lh5788sup1.cif

Structure factors: contains datablock(s) 1. DOI: 10.1107/S2056989015017272/lh57881sup2.hkl

Structure factors: contains datablock(s) 2. DOI: 10.1107/S2056989015017272/lh57882sup3.hkl

Structure factors: contains datablock(s) 3. DOI: 10.1107/S2056989015017272/lh57883sup4.hkl

Supporting information file. DOI: 10.1107/S2056989015017272/lh57881sup5.cml

CCDC references: 1424714, 1424713, 1424712

Additional supporting information:  crystallographic information; 3D view; checkCIF report

## Figures and Tables

**Table 1:** Hydrogen-bond geometry (Å, °) at 130 K[Chem scheme1]

*D*—H⋯*A*	*D*—H	H⋯*A*	*D*⋯*A*	*D*—H⋯*A*
N1—H1*A*⋯O4	0.88 (3)	2.03 (3)	2.886 (2)	166 (2)
N1—H1*B*⋯O1^i^	0.86 (3)	2.16 (3)	2.938 (2)	150 (2)
N1—H1*B*⋯O2^ii^	0.86 (3)	2.27 (3)	2.955 (2)	137 (2)
O2—H2⋯O4	0.81 (3)	1.77 (3)	2.5160 (16)	152 (4)
O2—H2⋯O3^iii^	0.81 (3)	2.57 (3)	3.0613 (17)	120 (3)
O4—H4⋯O2	0.82 (3)	1.82 (4)	2.5160 (16)	143 (4)
C7—H7*A*⋯O4^ii^	0.99	2.53	3.391 (2)	145
C10—H10*B*⋯Br2^iv^	0.99	2.90	3.8892 (17)	175

Symmetry codes: (i) 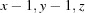; (ii) 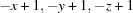; (iii) 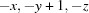; (iv) 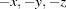.

**Table 2:** Hydrogen-bond geometry (Å, °) at 145 K[Chem scheme1]

*D*—H⋯*A*	*D*—H	H⋯*A*	*D*⋯*A*	*D*—H⋯*A*
N1—H1*A*⋯O4	0.86 (3)	2.04 (3)	2.888 (2)	166 (2)
N1—H1*B*⋯O1^i^	0.85 (3)	2.17 (3)	2.938 (2)	151 (2)
N1—H1*B*⋯O2^ii^	0.85 (3)	2.29 (3)	2.959 (2)	136 (2)
O2—H2⋯O4	0.82 (3)	1.77 (3)	2.5174 (16)	153 (4)
O2—H2⋯O3^iii^	0.82 (3)	2.58 (3)	3.0628 (17)	120 (3)
O4—H4⋯O2	0.82 (3)	1.79 (4)	2.5174 (16)	147 (4)
C7—H7*A*⋯O4^ii^	0.99	2.54	3.394 (2)	145
C10—H10*B*⋯Br2^iv^	0.99	2.90	3.8905 (17)	175

Symmetry codes: (i) 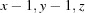; (ii) 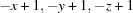; (iii) 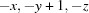; (iv) 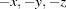.

**Table 3:** Hydrogen-bond geometry (Å, °) at 180 K[Chem scheme1]

*D*—H⋯*A*	*D*—H	H⋯*A*	*D*⋯*A*	*D*—H⋯*A*
N1—H1*A*⋯O4	0.89 (3)	2.02 (3)	2.890 (2)	167 (2)
N1—H1*B*⋯O1^i^	0.86 (3)	2.16 (3)	2.938 (2)	150 (2)
N1—H1*B*⋯O2^ii^	0.86 (3)	2.28 (3)	2.964 (2)	136 (2)
O2—H2⋯O4	0.82 (3)	1.79 (4)	2.5224 (16)	148 (5)
O2—H2⋯O3^iii^	0.82 (3)	2.55 (4)	3.0678 (18)	122 (4)
O4—H4⋯O2	0.82 (3)	1.80 (4)	2.5224 (16)	147 (4)
C7—H7*A*⋯O4^ii^	0.99	2.55	3.402 (2)	145
C10—H10*B*⋯Br2^iv^	0.99	2.91	3.8946 (17)	174

Symmetry codes: (i) 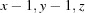; (ii) 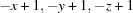; (iii) 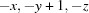; (iv) 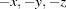.

**Table 4:** Experimental details

	130 K	145 K	180 K
Crystal data			
Chemical formula	C_4_H_10_NO^+^·C_6_HBr_2_O_4_^−^	C_4_H_10_NO^+^·C_6_HBr_2_O_4_^−^	C_4_H_10_NO^+^·C_6_HBr_2_O_4_^−^
*M*_r_	385.01	385.01	385.01
Crystal system, space group	Triclinic, *P* 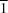	Triclinic, *P* 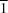	Triclinic, *P* 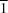
*a*, *b*, *c* (Å)	8.62046 (19), 9.2129 (2), 9.4257 (2)	8.62293 (18), 9.21849 (19), 9.4354 (2)	8.62824 (17), 9.23087 (18), 9.46007 (19)
α, β, γ (°)	93.5208 (7), 112.9139 (7), 115.9757 (7)	93.5239 (7), 112.9190 (7), 115.9777 (7)	93.5321 (7), 112.9738 (7), 115.9508 (7)
*V* (Å^3^)	595.05 (3)	596.13 (3)	598.67 (3)
*Z*	2	2	2
Radiation type	Mo *K*α	Mo *K*α	Mo *K*α
μ (mm^−1^)	6.84	6.83	6.80
Crystal size (mm)	0.40 × 0.34 × 0.18	0.40 × 0.34 × 0.18	0.40 × 0.34 × 0.18

Data collection			
Diffractometer	Rigaku R-AXIS RAPIDII	Rigaku R-AXIS RAPIDII	Rigaku R-AXIS RAPIDII
Absorption correction	Numerical (*NUMABS*; Higashi, 1999 ▸)	Numerical (*NUMABS*; Higashi, 1999 ▸)	Numerical (*NUMABS*; Higashi, 1999 ▸)
*T*_min_, *T*_max_	0.096, 0.292	0.098, 0.292	0.098, 0.294
No. of measured, independent and observed [*I* > 2σ(*I*)] reflections	18162, 3468, 3183	18176, 3473, 3181	18199, 3487, 3188
*R*_int_	0.026	0.028	0.026
(sin θ/λ)_max_ (Å^−1^)	0.704	0.704	0.703

Refinement			
*R*[*F* ^2^ > 2σ(*F* ^2^)], *wR*(*F* ^2^), *S*	0.017, 0.046, 1.14	0.018, 0.046, 1.10	0.019, 0.048, 1.09
No. of reflections	3468	3473	3487
No. of parameters	178	178	178
No. of restraints	2	2	2
H-atom treatment	H atoms treated by a mixture of independent and constrained refinement	H atoms treated by a mixture of independent and constrained refinement	H atoms treated by a mixture of independent and constrained refinement
Δρ_max_, Δρ_min_ (e Å^−3^)	0.50, −0.37	0.48, −0.44	0.59, −0.45

Computer programs: *RAPID-AUTO* (Rigaku, 2006 ▸), *SIR92* (Altomare *et al.*, 1994 ▸), *SHELXL2014/7* (Sheldrick, 2015 ▸), *ORTEP-3 for Windows* (Farrugia, 2012 ▸), *CrystalStructure* (Rigaku, 2010 ▸) and *PLATON* (Spek, 2009 ▸).

## supplementary crystallographic information

### (1) Morpholin-4-ium 2,5-dibromo-4-hydroxy-3,6-dioxocyclohexa-1,4-dien-1-olate Crystal data

**Table:**

C_4_H_10_NO^+^·C_6_HBr_2_O_4_^−^	*Z* = 2
*M**_r_* = 385.01	*F*(000) = 376.00
Triclinic, *P*1	*D*_x_ = 2.149 Mg m^−^^3^
Hall symbol: -P 1	Mo *K*α radiation, λ = 0.71075 Å
*a* = 8.62046 (19) Å	Cell parameters from 15996 reflections
*b* = 9.2129 (2) Å	θ = 3.0–30.1°
*c* = 9.4257 (2) Å	µ = 6.84 mm^−^^1^
α = 93.5208 (7)°	*T* = 130 K
β = 112.9139 (7)°	Block, brown
γ = 115.9757 (7)°	0.40 × 0.34 × 0.18 mm
*V* = 595.05 (3) Å^3^	

### (1) Morpholin-4-ium 2,5-dibromo-4-hydroxy-3,6-dioxocyclohexa-1,4-dien-1-olate Data collection

**Table:**

Rigaku R-AXIS RAPIDII diffractometer	3183 reflections with *I* > 2σ(*I*)
Detector resolution: 10.000 pixels mm^-1^	*R*_int_ = 0.026
ω scans	θ_max_ = 30.0°, θ_min_ = 3.0°
Absorption correction: numerical (*NUMABS*; Higashi, 1999)	*h* = −12→12
*T*_min_ = 0.096, *T*_max_ = 0.292	*k* = −12→12
18162 measured reflections	*l* = −13→13
3468 independent reflections	

### (1) Morpholin-4-ium 2,5-dibromo-4-hydroxy-3,6-dioxocyclohexa-1,4-dien-1-olate Refinement

**Table:**

Refinement on *F*^2^	2 restraints
Least-squares matrix: full	Hydrogen site location: mixed
*R*[*F*^2^ > 2σ(*F*^2^)] = 0.017	H atoms treated by a mixture of independent and constrained refinement
*wR*(*F*^2^) = 0.046	*w* = 1/[σ^2^(*F*_o_^2^) + (0.0195*P*)^2^ + 0.3723*P*] where *P* = (*F*_o_^2^ + 2*F*_c_^2^)/3
*S* = 1.14	(Δ/σ)_max_ = 0.002
3468 reflections	Δρ_max_ = 0.50 e Å^−^^3^
178 parameters	Δρ_min_ = −0.37 e Å^−^^3^

### (1) Morpholin-4-ium 2,5-dibromo-4-hydroxy-3,6-dioxocyclohexa-1,4-dien-1-olate Special details

**Table:**

Geometry. All e.s.d.'s (except the e.s.d. in the dihedral angle between two l.s. planes) are estimated using the full covariance matrix. The cell e.s.d.'s are taken into account individually in the estimation of e.s.d.'s in distances, angles and torsion angles; correlations between e.s.d.'s in cell parameters are only used when they are defined by crystal symmetry. An approximate (isotropic) treatment of cell e.s.d.'s is used for estimating e.s.d.'s involving l.s. planes.
Refinement. Reflections were merged by *SHELXL* according to the crystal class for the calculation of statistics and refinement.

### (1) Morpholin-4-ium 2,5-dibromo-4-hydroxy-3,6-dioxocyclohexa-1,4-dien-1-olate Fractional atomic coordinates and isotropic or equivalent isotropic displacement parameters (Å^2^)

**Table:**

	*x*	*y*	*z*	*U*_iso_*/*U*_eq_	Occ. (<1)
Br1	0.75783 (2)	0.69637 (2)	0.16055 (2)	0.01583 (4)	
Br2	−0.01546 (2)	0.16774 (2)	0.12333 (2)	0.01833 (4)	
O1	1.18246 (16)	0.98222 (14)	0.33171 (13)	0.0166 (2)	
O2	0.62026 (15)	0.76781 (13)	0.41041 (13)	0.0157 (2)	
H2	0.548 (5)	0.699 (4)	0.323 (3)	0.023*	0.49 (3)
O3	−0.34652 (16)	0.22816 (14)	−0.08651 (15)	0.0209 (2)	
O4	0.33771 (16)	0.50578 (13)	0.19213 (13)	0.0164 (2)	
H4	0.427 (5)	0.603 (3)	0.226 (5)	0.025*	0.51 (3)
O5	0.82461 (16)	0.26388 (15)	0.42254 (14)	0.0201 (2)	
N1	0.48737 (19)	0.29440 (17)	0.32853 (17)	0.0170 (2)	
C1	1.0924 (2)	0.98617 (17)	0.40452 (17)	0.0123 (2)	
C2	0.8902 (2)	0.86739 (17)	0.35299 (17)	0.0124 (2)	
C3	0.7974 (2)	0.87220 (17)	0.44093 (17)	0.0126 (2)	
C4	−0.1856 (2)	0.34950 (18)	−0.04567 (18)	0.0146 (3)	
C5	−0.0041 (2)	0.35821 (17)	0.05629 (17)	0.0139 (3)	
C6	0.1730 (2)	0.49677 (18)	0.10262 (17)	0.0145 (3)	
C7	0.6812 (2)	0.42756 (19)	0.45862 (19)	0.0176 (3)	
H7A	0.6639	0.4841	0.5393	0.021*	
H7B	0.7493	0.5130	0.4132	0.021*	
C8	0.8010 (2)	0.3481 (2)	0.53723 (19)	0.0188 (3)	
H8A	0.9304	0.4362	0.6229	0.023*	
H8B	0.7355	0.2669	0.5872	0.023*	
C9	0.6388 (2)	0.1307 (2)	0.3035 (2)	0.0206 (3)	
H9A	0.5750	0.0516	0.3557	0.025*	
H9B	0.6567	0.0681	0.2277	0.025*	
C10	0.5099 (2)	0.1976 (2)	0.21215 (19)	0.0196 (3)	
H10A	0.5690	0.2717	0.1543	0.023*	
H10B	0.3808	0.1032	0.1324	0.023*	
H1A	0.423 (3)	0.342 (3)	0.277 (3)	0.023 (5)*	
H1B	0.422 (3)	0.223 (3)	0.367 (3)	0.027 (6)*	

### (1) Morpholin-4-ium 2,5-dibromo-4-hydroxy-3,6-dioxocyclohexa-1,4-dien-1-olate Atomic displacement parameters (Å^2^)

**Table:**

	*U*^11^	*U*^22^	*U*^33^	*U*^12^	*U*^13^	*U*^23^
Br1	0.01430 (7)	0.01537 (7)	0.01367 (7)	0.00509 (6)	0.00595 (5)	0.00068 (5)
Br2	0.01566 (8)	0.01334 (7)	0.02186 (8)	0.00595 (6)	0.00607 (6)	0.00785 (6)
O1	0.0150 (5)	0.0183 (5)	0.0179 (5)	0.0075 (4)	0.0102 (4)	0.0046 (4)
O2	0.0100 (5)	0.0154 (5)	0.0166 (5)	0.0031 (4)	0.0059 (4)	0.0020 (4)
O3	0.0129 (5)	0.0152 (5)	0.0277 (6)	0.0040 (4)	0.0065 (4)	0.0072 (4)
O4	0.0122 (5)	0.0129 (5)	0.0182 (5)	0.0048 (4)	0.0036 (4)	0.0037 (4)
O5	0.0151 (5)	0.0241 (6)	0.0217 (5)	0.0117 (5)	0.0073 (4)	0.0036 (4)
N1	0.0139 (6)	0.0193 (6)	0.0223 (6)	0.0101 (5)	0.0096 (5)	0.0109 (5)
C1	0.0117 (6)	0.0115 (6)	0.0141 (6)	0.0063 (5)	0.0056 (5)	0.0052 (5)
C2	0.0109 (6)	0.0119 (6)	0.0120 (6)	0.0047 (5)	0.0044 (5)	0.0022 (5)
C3	0.0106 (6)	0.0122 (6)	0.0145 (6)	0.0064 (5)	0.0045 (5)	0.0049 (5)
C4	0.0152 (7)	0.0125 (6)	0.0155 (6)	0.0060 (5)	0.0080 (5)	0.0028 (5)
C5	0.0147 (6)	0.0115 (6)	0.0153 (6)	0.0062 (5)	0.0070 (5)	0.0048 (5)
C6	0.0158 (7)	0.0129 (6)	0.0133 (6)	0.0058 (5)	0.0072 (5)	0.0023 (5)
C7	0.0180 (7)	0.0161 (7)	0.0198 (7)	0.0090 (6)	0.0093 (6)	0.0052 (6)
C8	0.0170 (7)	0.0231 (7)	0.0167 (7)	0.0118 (6)	0.0064 (6)	0.0052 (6)
C9	0.0189 (7)	0.0171 (7)	0.0232 (8)	0.0099 (6)	0.0068 (6)	0.0032 (6)
C10	0.0174 (7)	0.0182 (7)	0.0179 (7)	0.0086 (6)	0.0043 (6)	0.0019 (6)

### (1) Morpholin-4-ium 2,5-dibromo-4-hydroxy-3,6-dioxocyclohexa-1,4-dien-1-olate Geometric parameters (Å, º)

**Table:**

Br1—C2	1.8807 (14)	C1—C3^i^	1.5265 (19)
Br2—C5	1.8767 (14)	C2—C3	1.368 (2)
O1—C1	1.2295 (18)	C4—C5	1.445 (2)
O2—C3	1.2953 (17)	C4—C6^ii^	1.520 (2)
O2—H2	0.81 (3)	C5—C6	1.362 (2)
O3—C4	1.2229 (18)	C7—C8	1.511 (2)
O4—C6	1.3002 (18)	C7—H7A	0.9900
O4—H4	0.82 (3)	C7—H7B	0.9900
O5—C8	1.4212 (19)	C8—H8A	0.9900
O5—C9	1.428 (2)	C8—H8B	0.9900
N1—C7	1.492 (2)	C9—C10	1.509 (2)
N1—C10	1.494 (2)	C9—H9A	0.9900
N1—H1A	0.88 (3)	C9—H9B	0.9900
N1—H1B	0.86 (3)	C10—H10A	0.9900
C1—C2	1.4407 (19)	C10—H10B	0.9900
			
C3—O2—H2	118 (3)	C5—C6—C4^ii^	120.03 (13)
C6—O4—H4	111 (3)	N1—C7—C8	109.20 (12)
C8—O5—C9	109.71 (12)	N1—C7—H7A	109.8
C7—N1—C10	110.91 (12)	C8—C7—H7A	109.8
C7—N1—H1A	109.1 (14)	N1—C7—H7B	109.8
C10—N1—H1A	108.4 (14)	C8—C7—H7B	109.8
C7—N1—H1B	111.0 (15)	H7A—C7—H7B	108.3
C10—N1—H1B	107.1 (15)	O5—C8—C7	110.61 (13)
H1A—N1—H1B	110 (2)	O5—C8—H8A	109.5
O1—C1—C2	124.43 (13)	C7—C8—H8A	109.5
O1—C1—C3^i^	117.51 (13)	O5—C8—H8B	109.5
C2—C1—C3^i^	118.06 (12)	C7—C8—H8B	109.5
C3—C2—C1	122.59 (13)	H8A—C8—H8B	108.1
C3—C2—Br1	120.39 (11)	O5—C9—C10	111.23 (13)
C1—C2—Br1	116.98 (10)	O5—C9—H9A	109.4
O2—C3—C2	127.82 (13)	C10—C9—H9A	109.4
O2—C3—C1^i^	112.91 (12)	O5—C9—H9B	109.4
C2—C3—C1^i^	119.28 (12)	C10—C9—H9B	109.4
O3—C4—C5	124.23 (14)	H9A—C9—H9B	108.0
O3—C4—C6^ii^	118.64 (13)	N1—C10—C9	108.70 (13)
C5—C4—C6^ii^	117.13 (12)	N1—C10—H10A	109.9
C6—C5—C4	122.84 (13)	C9—C10—H10A	109.9
C6—C5—Br2	119.19 (11)	N1—C10—H10B	109.9
C4—C5—Br2	117.97 (10)	C9—C10—H10B	109.9
O4—C6—C5	123.65 (14)	H10A—C10—H10B	108.3
O4—C6—C4^ii^	116.29 (13)		
			
O1—C1—C2—C3	177.29 (14)	C6^ii^—C4—C5—Br2	−178.24 (10)
C3^i^—C1—C2—C3	−3.3 (2)	C4—C5—C6—O4	−178.83 (14)
O1—C1—C2—Br1	−0.27 (19)	Br2—C5—C6—O4	−0.1 (2)
C3^i^—C1—C2—Br1	179.12 (9)	C4—C5—C6—C4^ii^	−0.5 (2)
C1—C2—C3—O2	−176.34 (14)	Br2—C5—C6—C4^ii^	178.22 (10)
Br1—C2—C3—O2	1.1 (2)	C10—N1—C7—C8	−54.86 (16)
C1—C2—C3—C1^i^	3.4 (2)	C9—O5—C8—C7	−62.32 (16)
Br1—C2—C3—C1^i^	−179.16 (9)	N1—C7—C8—O5	58.54 (17)
O3—C4—C5—C6	−178.49 (15)	C8—O5—C9—C10	62.50 (17)
C6^ii^—C4—C5—C6	0.5 (2)	C7—N1—C10—C9	54.40 (17)
O3—C4—C5—Br2	2.7 (2)	O5—C9—C10—N1	−58.02 (17)

Symmetry codes: (i) −*x*+2, −*y*+2, −*z*+1; (ii) −*x*, −*y*+1, −*z*.

### (1) Morpholin-4-ium 2,5-dibromo-4-hydroxy-3,6-dioxocyclohexa-1,4-dien-1-olate Hydrogen-bond geometry (Å, º)

**Table:**

*D*—H···*A*	*D*—H	H···*A*	*D*···*A*	*D*—H···*A*
N1—H1*A*···O4	0.88 (3)	2.03 (3)	2.886 (2)	166 (2)
N1—H1*B*···O1^iii^	0.86 (3)	2.16 (3)	2.938 (2)	150 (2)
N1—H1*B*···O2^iv^	0.86 (3)	2.27 (3)	2.955 (2)	137 (2)
O2—H2···O4	0.81 (3)	1.77 (3)	2.5160 (16)	152 (4)
O2—H2···O3^ii^	0.81 (3)	2.57 (3)	3.0613 (17)	120 (3)
O4—H4···O2	0.82 (3)	1.82 (4)	2.5160 (16)	143 (4)
C7—H7*A*···O4^iv^	0.99	2.53	3.391 (2)	145
C10—H10*B*···Br2^v^	0.99	2.90	3.8892 (17)	175

Symmetry codes: (ii) −*x*, −*y*+1, −*z*; (iii) *x*−1, *y*−1, *z*; (iv) −*x*+1, −*y*+1, −*z*+1; (v) −*x*, −*y*, −*z*.

### (2) Crystal data

**Table:**

C_4_H_10_NO^+^·C_6_HBr_2_O_4_^−^	*Z* = 2
*M**_r_* = 385.01	*F*(000) = 376.00
Triclinic, *P*1	*D*_x_ = 2.145 Mg m^−^^3^
Hall symbol: -P 1	Mo *K*α radiation, λ = 0.71075 Å
*a* = 8.62293 (18) Å	Cell parameters from 15916 reflections
*b* = 9.21849 (19) Å	θ = 3.0–30.1°
*c* = 9.4354 (2) Å	µ = 6.83 mm^−^^1^
α = 93.5239 (7)°	*T* = 145 K
β = 112.9190 (7)°	Block, brown
γ = 115.9777 (7)°	0.40 × 0.34 × 0.18 mm
*V* = 596.13 (3) Å^3^	

### (2) Data collection

**Table:**

Rigaku R-AXIS RAPIDII diffractometer	3181 reflections with *I* > 2σ(*I*)
Detector resolution: 10.000 pixels mm^-1^	*R*_int_ = 0.028
ω scans	θ_max_ = 30.0°, θ_min_ = 3.0°
Absorption correction: numerical (*NUMABS*; Higashi, 1999)	*h* = −12→12
*T*_min_ = 0.098, *T*_max_ = 0.292	*k* = −12→12
18176 measured reflections	*l* = −13→13
3473 independent reflections	

### (2) Refinement

**Table:**

Refinement on *F*^2^	2 restraints
Least-squares matrix: full	Hydrogen site location: mixed
*R*[*F*^2^ > 2σ(*F*^2^)] = 0.018	H atoms treated by a mixture of independent and constrained refinement
*wR*(*F*^2^) = 0.046	*w* = 1/[σ^2^(*F*_o_^2^) + (0.020*P*)^2^ + 0.3611*P*] where *P* = (*F*_o_^2^ + 2*F*_c_^2^)/3
*S* = 1.10	(Δ/σ)_max_ = 0.001
3473 reflections	Δρ_max_ = 0.48 e Å^−^^3^
178 parameters	Δρ_min_ = −0.44 e Å^−^^3^

### (2) Special details

**Table:**

Geometry. All e.s.d.'s (except the e.s.d. in the dihedral angle between two l.s. planes) are estimated using the full covariance matrix. The cell e.s.d.'s are taken into account individually in the estimation of e.s.d.'s in distances, angles and torsion angles; correlations between e.s.d.'s in cell parameters are only used when they are defined by crystal symmetry. An approximate (isotropic) treatment of cell e.s.d.'s is used for estimating e.s.d.'s involving l.s. planes.
Refinement. Reflections were merged by *SHELXL* according to the crystal class for the calculation of statistics and refinement.

### (2) Fractional atomic coordinates and isotropic or equivalent isotropic displacement parameters (Å^2^)

**Table:**

	*x*	*y*	*z*	*U*_iso_*/*U*_eq_	Occ. (<1)
Br1	0.75812 (2)	0.69658 (2)	0.16087 (2)	0.01737 (4)	
Br2	−0.01570 (2)	0.16791 (2)	0.12324 (2)	0.02003 (5)	
O1	1.18243 (15)	0.98258 (14)	0.33190 (13)	0.0179 (2)	
O2	0.62059 (15)	0.76777 (13)	0.41035 (13)	0.0172 (2)	
H2	0.547 (5)	0.698 (4)	0.323 (3)	0.026*	0.52 (3)
O3	−0.34653 (16)	0.22841 (14)	−0.08661 (15)	0.0227 (2)	
O4	0.33774 (15)	0.50586 (13)	0.19208 (13)	0.0175 (2)	
H4	0.430 (5)	0.603 (3)	0.231 (5)	0.026*	0.48 (3)
O5	0.82422 (16)	0.26379 (15)	0.42261 (14)	0.0219 (2)	
N1	0.48734 (19)	0.29444 (17)	0.32852 (17)	0.0183 (2)	
C1	1.0922 (2)	0.98623 (17)	0.40454 (17)	0.0131 (2)	
C2	0.8905 (2)	0.86761 (17)	0.35306 (17)	0.0138 (2)	
C3	0.7975 (2)	0.87207 (17)	0.44087 (17)	0.0133 (2)	
C4	−0.1856 (2)	0.34979 (18)	−0.04558 (17)	0.0156 (3)	
C5	−0.0041 (2)	0.35843 (17)	0.05629 (17)	0.0149 (3)	
C6	0.1731 (2)	0.49685 (18)	0.10257 (17)	0.0154 (3)	
C7	0.6812 (2)	0.42735 (19)	0.45871 (19)	0.0191 (3)	
H7A	0.6639	0.4839	0.5393	0.023*	
H7B	0.7495	0.5128	0.4135	0.023*	
C8	0.8006 (2)	0.3479 (2)	0.53709 (19)	0.0204 (3)	
H8A	0.9300	0.4358	0.6228	0.025*	
H8B	0.7349	0.2667	0.5868	0.025*	
C9	0.6387 (2)	0.1307 (2)	0.3035 (2)	0.0227 (3)	
H9A	0.5748	0.0515	0.3556	0.027*	
H9B	0.6567	0.0683	0.2278	0.027*	
C10	0.5097 (2)	0.1977 (2)	0.21235 (19)	0.0212 (3)	
H10A	0.5687	0.2716	0.1545	0.025*	
H10B	0.3806	0.1034	0.1327	0.025*	
H1A	0.425 (3)	0.342 (3)	0.278 (3)	0.024 (5)*	
H1B	0.422 (3)	0.224 (3)	0.365 (3)	0.030 (6)*	

### (2) Atomic displacement parameters (Å^2^)

**Table:**

	*U*^11^	*U*^22^	*U*^33^	*U*^12^	*U*^13^	*U*^23^
Br1	0.01574 (7)	0.01685 (7)	0.01495 (7)	0.00556 (6)	0.00658 (5)	0.00055 (5)
Br2	0.01706 (8)	0.01455 (7)	0.02405 (8)	0.00653 (6)	0.00668 (6)	0.00865 (6)
O1	0.0161 (5)	0.0199 (5)	0.0195 (5)	0.0081 (4)	0.0110 (4)	0.0050 (4)
O2	0.0108 (5)	0.0164 (5)	0.0184 (5)	0.0030 (4)	0.0064 (4)	0.0015 (4)
O3	0.0136 (5)	0.0158 (5)	0.0308 (6)	0.0039 (4)	0.0068 (5)	0.0081 (4)
O4	0.0128 (5)	0.0138 (5)	0.0195 (5)	0.0051 (4)	0.0035 (4)	0.0038 (4)
O5	0.0167 (5)	0.0258 (6)	0.0240 (6)	0.0130 (5)	0.0079 (4)	0.0038 (5)
N1	0.0146 (6)	0.0210 (6)	0.0244 (7)	0.0108 (5)	0.0105 (5)	0.0118 (5)
C1	0.0124 (6)	0.0127 (6)	0.0150 (6)	0.0069 (5)	0.0061 (5)	0.0057 (5)
C2	0.0124 (6)	0.0134 (6)	0.0134 (6)	0.0056 (5)	0.0051 (5)	0.0025 (5)
C3	0.0113 (6)	0.0125 (6)	0.0151 (6)	0.0064 (5)	0.0046 (5)	0.0049 (5)
C4	0.0168 (7)	0.0131 (6)	0.0161 (6)	0.0063 (5)	0.0083 (5)	0.0028 (5)
C5	0.0156 (6)	0.0123 (6)	0.0161 (6)	0.0067 (5)	0.0071 (5)	0.0047 (5)
C6	0.0166 (7)	0.0139 (6)	0.0139 (6)	0.0060 (5)	0.0076 (5)	0.0021 (5)
C7	0.0195 (7)	0.0172 (7)	0.0223 (7)	0.0095 (6)	0.0107 (6)	0.0059 (6)
C8	0.0187 (7)	0.0251 (7)	0.0177 (7)	0.0129 (6)	0.0066 (6)	0.0051 (6)
C9	0.0209 (7)	0.0185 (7)	0.0255 (8)	0.0108 (6)	0.0073 (6)	0.0028 (6)
C10	0.0185 (7)	0.0206 (7)	0.0189 (7)	0.0093 (6)	0.0047 (6)	0.0022 (6)

### (2) Geometric parameters (Å, º)

**Table:**

Br1—C2	1.8807 (14)	C1—C3^i^	1.5282 (19)
Br2—C5	1.8775 (14)	C2—C3	1.3684 (19)
O1—C1	1.2298 (17)	C4—C5	1.446 (2)
O2—C3	1.2937 (17)	C4—C6^ii^	1.518 (2)
O2—H2	0.82 (3)	C5—C6	1.363 (2)
O3—C4	1.2231 (18)	C7—C8	1.509 (2)
O4—C6	1.2997 (18)	C7—H7A	0.9900
O4—H4	0.82 (3)	C7—H7B	0.9900
O5—C8	1.4201 (19)	C8—H8A	0.9900
O5—C9	1.428 (2)	C8—H8B	0.9900
N1—C7	1.492 (2)	C9—C10	1.510 (2)
N1—C10	1.493 (2)	C9—H9A	0.9900
N1—H1A	0.86 (3)	C9—H9B	0.9900
N1—H1B	0.85 (3)	C10—H10A	0.9900
C1—C2	1.4377 (19)	C10—H10B	0.9900
			
C3—O2—H2	118 (3)	C5—C6—C4^ii^	119.95 (13)
C6—O4—H4	113 (3)	N1—C7—C8	109.23 (12)
C8—O5—C9	109.77 (12)	N1—C7—H7A	109.8
C7—N1—C10	110.93 (12)	C8—C7—H7A	109.8
C7—N1—H1A	108.5 (14)	N1—C7—H7B	109.8
C10—N1—H1A	108.6 (14)	C8—C7—H7B	109.8
C7—N1—H1B	111.9 (16)	H7A—C7—H7B	108.3
C10—N1—H1B	106.6 (15)	O5—C8—C7	110.62 (13)
H1A—N1—H1B	110 (2)	O5—C8—H8A	109.5
O1—C1—C2	124.48 (13)	C7—C8—H8A	109.5
O1—C1—C3^i^	117.39 (12)	O5—C8—H8B	109.5
C2—C1—C3^i^	118.13 (12)	C7—C8—H8B	109.5
C3—C2—C1	122.62 (13)	H8A—C8—H8B	108.1
C3—C2—Br1	120.30 (11)	O5—C9—C10	111.19 (13)
C1—C2—Br1	117.03 (10)	O5—C9—H9A	109.4
O2—C3—C2	127.87 (13)	C10—C9—H9A	109.4
O2—C3—C1^i^	112.96 (12)	O5—C9—H9B	109.4
C2—C3—C1^i^	119.17 (12)	C10—C9—H9B	109.4
O3—C4—C5	124.23 (13)	H9A—C9—H9B	108.0
O3—C4—C6^ii^	118.58 (13)	N1—C10—C9	108.74 (13)
C5—C4—C6^ii^	117.19 (12)	N1—C10—H10A	109.9
C6—C5—C4	122.86 (13)	C9—C10—H10A	109.9
C6—C5—Br2	119.20 (11)	N1—C10—H10B	109.9
C4—C5—Br2	117.92 (10)	C9—C10—H10B	109.9
O4—C6—C5	123.68 (13)	H10A—C10—H10B	108.3
O4—C6—C4^ii^	116.36 (12)		
			
O1—C1—C2—C3	177.25 (14)	C6^ii^—C4—C5—Br2	−178.23 (10)
C3^i^—C1—C2—C3	−3.4 (2)	C4—C5—C6—O4	−178.92 (13)
O1—C1—C2—Br1	−0.21 (19)	Br2—C5—C6—O4	−0.2 (2)
C3^i^—C1—C2—Br1	179.18 (9)	C4—C5—C6—C4^ii^	−0.6 (2)
C1—C2—C3—O2	−176.36 (13)	Br2—C5—C6—C4^ii^	178.19 (10)
Br1—C2—C3—O2	1.0 (2)	C10—N1—C7—C8	−54.81 (16)
C1—C2—C3—C1^i^	3.4 (2)	C9—O5—C8—C7	−62.38 (16)
Br1—C2—C3—C1^i^	−179.22 (9)	N1—C7—C8—O5	58.53 (17)
O3—C4—C5—C6	−178.65 (15)	C8—O5—C9—C10	62.43 (17)
C6^ii^—C4—C5—C6	0.5 (2)	C7—N1—C10—C9	54.27 (17)
O3—C4—C5—Br2	2.6 (2)	O5—C9—C10—N1	−57.89 (18)

Symmetry codes: (i) −*x*+2, −*y*+2, −*z*+1; (ii) −*x*, −*y*+1, −*z*.

### (2) Hydrogen-bond geometry (Å, º)

**Table:**

*D*—H···*A*	*D*—H	H···*A*	*D*···*A*	*D*—H···*A*
N1—H1*A*···O4	0.86 (3)	2.04 (3)	2.888 (2)	166 (2)
N1—H1*B*···O1^iii^	0.85 (3)	2.17 (3)	2.938 (2)	151 (2)
N1—H1*B*···O2^iv^	0.85 (3)	2.29 (3)	2.959 (2)	136 (2)
O2—H2···O4	0.82 (3)	1.77 (3)	2.5174 (16)	153 (4)
O2—H2···O3^ii^	0.82 (3)	2.58 (3)	3.0628 (17)	120 (3)
O4—H4···O2	0.82 (3)	1.79 (4)	2.5174 (16)	147 (4)
C7—H7*A*···O4^iv^	0.99	2.54	3.394 (2)	145
C10—H10*B*···Br2^v^	0.99	2.90	3.8905 (17)	175

Symmetry codes: (ii) −*x*, −*y*+1, −*z*; (iii) *x*−1, *y*−1, *z*; (iv) −*x*+1, −*y*+1, −*z*+1; (v) −*x*, −*y*, −*z*.

### (3) Crystal data

**Table:**

C_4_H_10_NO^+^·C_6_HBr_2_O_4_^−^	*Z* = 2
*M**_r_* = 385.01	*F*(000) = 376.00
Triclinic, *P*1	*D*_x_ = 2.136 Mg m^−^^3^
Hall symbol: -P 1	Mo *K*α radiation, λ = 0.71075 Å
*a* = 8.62824 (17) Å	Cell parameters from 15838 reflections
*b* = 9.23087 (18) Å	θ = 3.0–30.1°
*c* = 9.46007 (19) Å	µ = 6.80 mm^−^^1^
α = 93.5321 (7)°	*T* = 180 K
β = 112.9738 (7)°	Block, brown
γ = 115.9508 (7)°	0.40 × 0.34 × 0.18 mm
*V* = 598.67 (3) Å^3^	

### (3) Data collection

**Table:**

Rigaku R-AXIS RAPIDII diffractometer	3188 reflections with *I* > 2σ(*I*)
Detector resolution: 10.000 pixels mm^-1^	*R*_int_ = 0.026
ω scans	θ_max_ = 30.0°
Absorption correction: numerical (*NUMABS*; Higashi, 1999)	*h* = −12→12
*T*_min_ = 0.098, *T*_max_ = 0.294	*k* = −12→12
18199 measured reflections	*l* = −13→13
3487 independent reflections	

### (3) Refinement

**Table:**

Refinement on *F*^2^	2 restraints
Least-squares matrix: full	Hydrogen site location: mixed
*R*[*F*^2^ > 2σ(*F*^2^)] = 0.019	H atoms treated by a mixture of independent and constrained refinement
*wR*(*F*^2^) = 0.048	*w* = 1/[σ^2^(*F*_o_^2^) + (0.0216*P*)^2^ + 0.3489*P*] where *P* = (*F*_o_^2^ + 2*F*_c_^2^)/3
*S* = 1.09	(Δ/σ)_max_ = 0.001
3487 reflections	Δρ_max_ = 0.59 e Å^−^^3^
178 parameters	Δρ_min_ = −0.45 e Å^−^^3^

### (3) Special details

**Table:**

Geometry. All e.s.d.'s (except the e.s.d. in the dihedral angle between two l.s. planes) are estimated using the full covariance matrix. The cell e.s.d.'s are taken into account individually in the estimation of e.s.d.'s in distances, angles and torsion angles; correlations between e.s.d.'s in cell parameters are only used when they are defined by crystal symmetry. An approximate (isotropic) treatment of cell e.s.d.'s is used for estimating e.s.d.'s involving l.s. planes.
Refinement. Reflections were merged by *SHELXL* according to the crystal class for the calculation of statistics and refinement.

### (3) Fractional atomic coordinates and isotropic or equivalent isotropic displacement parameters (Å^2^)

**Table:**

	*x*	*y*	*z*	*U*_iso_*/*U*_eq_	Occ. (<1)
Br1	0.75874 (2)	0.69709 (2)	0.16160 (2)	0.02126 (5)	
Br2	−0.01629 (2)	0.16834 (2)	0.12298 (2)	0.02440 (5)	
O1	1.18225 (16)	0.98340 (14)	0.33207 (13)	0.0212 (2)	
O2	0.62107 (15)	0.76749 (14)	0.41043 (13)	0.0205 (2)	
H2	0.550 (6)	0.702 (5)	0.321 (3)	0.031*	0.50 (3)
O3	−0.34638 (16)	0.22923 (14)	−0.08655 (16)	0.0275 (2)	
O4	0.33726 (16)	0.50565 (14)	0.19202 (13)	0.0209 (2)	
H4	0.426 (5)	0.604 (3)	0.231 (5)	0.031*	0.50 (3)
O5	0.82325 (17)	0.26349 (16)	0.42251 (15)	0.0266 (2)	
N1	0.48719 (19)	0.29464 (18)	0.32897 (17)	0.0216 (3)	
C1	1.0921 (2)	0.98669 (17)	0.40464 (17)	0.0155 (2)	
C2	0.8904 (2)	0.86771 (17)	0.35338 (17)	0.0158 (2)	
C3	0.7977 (2)	0.87190 (17)	0.44099 (17)	0.0157 (2)	
C4	−0.1853 (2)	0.35010 (18)	−0.04556 (18)	0.0185 (3)	
C5	−0.0045 (2)	0.35849 (17)	0.05607 (17)	0.0175 (3)	
C6	0.1728 (2)	0.49667 (18)	0.10245 (17)	0.0180 (3)	
C7	0.6809 (2)	0.42714 (19)	0.4585 (2)	0.0227 (3)	
H7A	0.6639	0.4840	0.5389	0.027*	
H7B	0.7492	0.5122	0.4133	0.027*	
C8	0.7997 (2)	0.3474 (2)	0.53683 (19)	0.0250 (3)	
H8A	0.9291	0.4350	0.6226	0.030*	
H8B	0.7337	0.2662	0.5861	0.030*	
C9	0.6381 (2)	0.1310 (2)	0.3036 (2)	0.0272 (3)	
H9A	0.5742	0.0517	0.3552	0.033*	
H9B	0.6562	0.0690	0.2279	0.033*	
C10	0.5094 (2)	0.1980 (2)	0.21303 (19)	0.0252 (3)	
H10A	0.5683	0.2719	0.1554	0.030*	
H10B	0.3803	0.1040	0.1335	0.030*	
H1A	0.424 (3)	0.344 (3)	0.277 (2)	0.025 (5)*	
H1B	0.422 (3)	0.223 (3)	0.367 (3)	0.026 (5)*	

### (3) Atomic displacement parameters (Å^2^)

**Table:**

	*U*^11^	*U*^22^	*U*^33^	*U*^12^	*U*^13^	*U*^23^
Br1	0.01905 (8)	0.02063 (8)	0.01805 (7)	0.00654 (6)	0.00783 (6)	0.00043 (5)
Br2	0.02061 (8)	0.01771 (7)	0.02936 (9)	0.00800 (6)	0.00791 (6)	0.01079 (6)
O1	0.0192 (5)	0.0234 (5)	0.0227 (5)	0.0093 (4)	0.0131 (4)	0.0057 (4)
O2	0.0128 (5)	0.0201 (5)	0.0215 (5)	0.0035 (4)	0.0076 (4)	0.0019 (4)
O3	0.0161 (5)	0.0194 (5)	0.0374 (7)	0.0049 (4)	0.0080 (5)	0.0101 (5)
O4	0.0151 (5)	0.0166 (5)	0.0230 (5)	0.0061 (4)	0.0039 (4)	0.0046 (4)
O5	0.0192 (5)	0.0314 (6)	0.0295 (6)	0.0151 (5)	0.0094 (5)	0.0045 (5)
N1	0.0171 (6)	0.0245 (6)	0.0290 (7)	0.0129 (5)	0.0123 (5)	0.0141 (6)
C1	0.0148 (6)	0.0147 (6)	0.0180 (6)	0.0076 (5)	0.0079 (5)	0.0069 (5)
C2	0.0141 (6)	0.0144 (6)	0.0159 (6)	0.0057 (5)	0.0062 (5)	0.0027 (5)
C3	0.0133 (6)	0.0155 (6)	0.0180 (6)	0.0076 (5)	0.0064 (5)	0.0061 (5)
C4	0.0181 (7)	0.0155 (6)	0.0197 (6)	0.0065 (5)	0.0092 (5)	0.0034 (5)
C5	0.0175 (6)	0.0141 (6)	0.0192 (6)	0.0073 (5)	0.0078 (5)	0.0053 (5)
C6	0.0188 (7)	0.0164 (6)	0.0166 (6)	0.0071 (5)	0.0086 (5)	0.0030 (5)
C7	0.0231 (7)	0.0208 (7)	0.0259 (7)	0.0111 (6)	0.0129 (6)	0.0066 (6)
C8	0.0219 (7)	0.0308 (8)	0.0218 (7)	0.0150 (7)	0.0080 (6)	0.0061 (6)
C9	0.0250 (8)	0.0217 (7)	0.0317 (8)	0.0129 (7)	0.0095 (7)	0.0036 (6)
C10	0.0218 (7)	0.0243 (7)	0.0220 (7)	0.0108 (6)	0.0051 (6)	0.0025 (6)

### (3) Geometric parameters (Å, º)

**Table:**

Br1—C2	1.8803 (14)	C1—C3^i^	1.5283 (19)
Br2—C5	1.8769 (14)	C2—C3	1.367 (2)
O1—C1	1.2299 (17)	C4—C5	1.442 (2)
O2—C3	1.2931 (17)	C4—C6^ii^	1.519 (2)
O2—H2	0.82 (3)	C5—C6	1.363 (2)
O3—C4	1.2229 (18)	C7—C8	1.509 (2)
O4—C6	1.2997 (18)	C7—H7A	0.9900
O4—H4	0.82 (3)	C7—H7B	0.9900
O5—C8	1.420 (2)	C8—H8A	0.9900
O5—C9	1.425 (2)	C8—H8B	0.9900
N1—C7	1.490 (2)	C9—C10	1.508 (2)
N1—C10	1.493 (2)	C9—H9A	0.9900
N1—H1A	0.89 (3)	C9—H9B	0.9900
N1—H1B	0.86 (3)	C10—H10A	0.9900
C1—C2	1.4394 (19)	C10—H10B	0.9900
			
C3—O2—H2	117 (3)	C5—C6—C4^ii^	119.94 (13)
C6—O4—H4	111 (3)	N1—C7—C8	109.14 (13)
C8—O5—C9	109.83 (12)	N1—C7—H7A	109.9
C7—N1—C10	110.85 (12)	C8—C7—H7A	109.9
C7—N1—H1A	108.4 (14)	N1—C7—H7B	109.9
C10—N1—H1A	108.2 (14)	C8—C7—H7B	109.9
C7—N1—H1B	111.2 (14)	H7A—C7—H7B	108.3
C10—N1—H1B	106.4 (14)	O5—C8—C7	110.51 (13)
H1A—N1—H1B	112 (2)	O5—C8—H8A	109.5
O1—C1—C2	124.51 (13)	C7—C8—H8A	109.5
O1—C1—C3^i^	117.41 (12)	O5—C8—H8B	109.5
C2—C1—C3^i^	118.08 (12)	C7—C8—H8B	109.5
C3—C2—C1	122.63 (13)	H8A—C8—H8B	108.1
C3—C2—Br1	120.43 (11)	O5—C9—C10	111.18 (13)
C1—C2—Br1	116.90 (10)	O5—C9—H9A	109.4
O2—C3—C2	127.81 (13)	C10—C9—H9A	109.4
O2—C3—C1^i^	112.98 (12)	O5—C9—H9B	109.4
C2—C3—C1^i^	119.21 (12)	C10—C9—H9B	109.4
O3—C4—C5	124.27 (14)	H9A—C9—H9B	108.0
O3—C4—C6^ii^	118.48 (14)	N1—C10—C9	108.84 (13)
C5—C4—C6^ii^	117.24 (12)	N1—C10—H10A	109.9
C6—C5—C4	122.82 (13)	C9—C10—H10A	109.9
C6—C5—Br2	119.20 (11)	N1—C10—H10B	109.9
C4—C5—Br2	117.98 (10)	C9—C10—H10B	109.9
O4—C6—C5	123.69 (14)	H10A—C10—H10B	108.3
O4—C6—C4^ii^	116.35 (13)		
			
O1—C1—C2—C3	177.30 (14)	C6^ii^—C4—C5—Br2	−178.27 (10)
C3^i^—C1—C2—C3	−3.3 (2)	C4—C5—C6—O4	−178.95 (14)
O1—C1—C2—Br1	−0.20 (19)	Br2—C5—C6—O4	−0.2 (2)
C3^i^—C1—C2—Br1	179.24 (9)	C4—C5—C6—C4^ii^	−0.5 (2)
C1—C2—C3—O2	−176.48 (14)	Br2—C5—C6—C4^ii^	178.25 (10)
Br1—C2—C3—O2	0.9 (2)	C10—N1—C7—C8	−55.03 (17)
C1—C2—C3—C1^i^	3.3 (2)	C9—O5—C8—C7	−62.46 (17)
Br1—C2—C3—C1^i^	−179.29 (9)	N1—C7—C8—O5	58.77 (17)
O3—C4—C5—C6	−178.51 (15)	C8—O5—C9—C10	62.31 (18)
C6^ii^—C4—C5—C6	0.5 (2)	C7—N1—C10—C9	54.35 (17)
O3—C4—C5—Br2	2.7 (2)	O5—C9—C10—N1	−57.76 (19)

Symmetry codes: (i) −*x*+2, −*y*+2, −*z*+1; (ii) −*x*, −*y*+1, −*z*.

### (3) Hydrogen-bond geometry (Å, º)

**Table:**

*D*—H···*A*	*D*—H	H···*A*	*D*···*A*	*D*—H···*A*
N1—H1*A*···O4	0.89 (3)	2.02 (3)	2.890 (2)	167 (2)
N1—H1*B*···O1^iii^	0.86 (3)	2.16 (3)	2.938 (2)	150 (2)
N1—H1*B*···O2^iv^	0.86 (3)	2.28 (3)	2.964 (2)	136 (2)
O2—H2···O4	0.82 (3)	1.79 (4)	2.5224 (16)	148 (5)
O2—H2···O3^ii^	0.82 (3)	2.55 (4)	3.0678 (18)	122 (4)
O4—H4···O2	0.82 (3)	1.80 (4)	2.5224 (16)	147 (4)
C7—H7*A*···O4^iv^	0.99	2.55	3.402 (2)	145
C10—H10*B*···Br2^v^	0.99	2.91	3.8946 (17)	174

Symmetry codes: (ii) −*x*, −*y*+1, −*z*; (iii) *x*−1, *y*−1, *z*; (iv) −*x*+1, −*y*+1, −*z*+1; (v) −*x*, −*y*, −*z*.
